# Splenic Artery Aneurysm Following Endoscopic Ultrasound-Guided Fine-Needle Aspiration: A Rare Complication Presenting with Delayed Massive Gastrointestinal Bleeding

**DOI:** 10.5152/tjg.2025.25457

**Published:** 2025-10-19

**Authors:** Hüseyin Aykut, Baver Ordu, Berkay Dertsiz, Mehmet Ali Saruhan, Gupse Adalı

**Affiliations:** Department of Gastroenterology, University of Health Sciences, Ümraniye Training and Research Hospital, İstanbul, Türkiye

Dear Editor,

Splenic artery aneurysm (SAA) is a rare clinical condition defined as abnormal dilation of the splenic artery exceeding 1 cm in diameter.^1^ It is the third most common type among intra-abdominal aneurysms.^2^ Recognized risk factors for SAA include trauma, portal hypertension, pregnancy (related to hemodynamic and hormonal changes), connective tissue disorders (such as Ehlers-Danlos syndrome, Marfan syndrome, etc.), liver transplantation, and atherosclerosis.^1^^,^^3^ Prevalence is higher after the fifth decade and among women, with a female-to-male ratio of 4 : 1. Patients are often asymptomatic; however, as the size of the aneurysm increases (>2 cm), the likelihood of symptom development and the risk of complications also rise. The most severe complication is spontaneous aneurysm rupture, which may manifest as abrupt, intense abdominal pain and hemodynamic instability.^4^ The spontaneous rupture of SAA is associated with a significant mortality risk, though roughly 2%-10% of patients present clinical signs of rupture at the time of initial diagnosis. The aneurysm may penetrate into the lumen of an adjacent visceral organ it contacts, leading to massive gastrointestinal (GI) hemorrhage.^5^

A 75-year-old female patient with a medical history of asthma arrived at the emergency department with hematemesis. Her vital signs showed hypotension and tachycardia. The digital rectal examination indicated the presence of melena. The laboratory results were as follows: white blood cell count: 9320/mm^3^, hemoglobin: 7.9 g/dL, platelet count: 232 000/mm^3^, C-reactive protein: 19 mg/L, alanine aminotransferase: 11 U/L, aspartate aminotransferase: 14 U/L, blood urea nitrogen: 33 mg/dL, creatinine: 0.62 mg/dL, sodium: 137 mmol/L, potassium: 3.9 mmol/L. An initial gastroscopy following the red blood cell transfusion failed to reveal an active bleeding source in the stomach. However, due to persistent melena and an ongoing fall in hemoglobin levels, a second gastroscopy was performed, revealing fresh blood in the stomach. A suspicious erosive lesion was identified on the posterior aspect of the greater curvature of the gastric corpus, and hemostatic clipping was applied (Figure 1A). Despite this intervention, active bleeding continued, necessitating a third endoscopy, which revealed another erosive area with irregular mucosa in the cardia (Figure 1B). Hemostatic clipping was attempted; however, due to the rigidity of the tissue, it was unsuccessful, and the clip eventually fell into the fundus. Due to recurrent massive GI bleeding episodes, a computed tomography angiography (CTA) was performed to determine the bleeding source. Imaging revealed two cystic lesions in the pancreatic head and body, the largest measuring 18 × 14 mm, consistent with branch-duct intraductal papillary mucinous neoplasm. Additionally, aneurysmatic dilatations were detected in the abdominal aorta (38 mm) and the mid-section of the splenic artery (22 mm) (Figure 2A).

The detailed history revealed that the patient had undergone endoscopic ultrasound-guided fine-needle aspiration (EUS-FNA) approximately three months ago due to a cyst in the body of the pancreas. Following this procedure, she experienced severe stabbing abdominal pain, leading to multiple emergency visits. In the cross-sectional imaging performed during this period, a hematoma adjacent to the SAA was detected, and this hematoma was observed to regress over time. Therefore, it was followed conservatively without intervention. Current CTA findings demonstrated close proximity between the SAA and the gastric cardia, raising suspicion of aneurysmal rupture in the stomach (Figure 2B). Given the ongoing massive hemorrhagic episodes, the interventional radiology team was consulted, and coil embolization of the SAA was performed. Post embolization, the patient experienced no further episodes of GI bleeding. Follow-up imaging confirmed complete thrombosis of the aneurysm, with no residual blood flow (Figure 2C). A follow-up gastroscopy revealed a 2 cm exudative ulcer at the site previously identified as an erosive lesion in the cardia, consistent with post-embolization alterations (Figure 3A). The patient was discharged without further GI bleeding episodes, and follow-up endoscopy demonstrated near-complete healing of the ulcerated region (Figure 3B). Written informed consent was obtained from the patient for this case report.

Although rare, aneurysms should be considered in patients presenting with massive GI bleeding, especially when multiple endoscopic evaluations fail to identify a bleeding source. Timely diagnosis is critical, as aneurysm rupture can result in life-threatening hemorrhagic events. We present a rare case of a SAA likely induced by a previous EUS-FNA, which resulted in recurrent severe GI hemorrhage. The temporal relationship between the procedure and symptom onset, supported by imaging findings, indicates a causal association. There is a high probability that the incidence of uncommon complications, such as vascular aneurysms, will rise as a result of the increasing prevalence of EUS-guided invasive procedures. Clinicians should maintain a high index of suspicion for aneurysms in patients presenting with unexplained GI hemorrhage and a pertinent procedural history. Early diagnosis and intervention require a comprehensive clinical history, strong clinical suspicion, and appropriate imaging techniques, which together enhance patient outcomes.

## Figures and Tables

**Figure 1.**
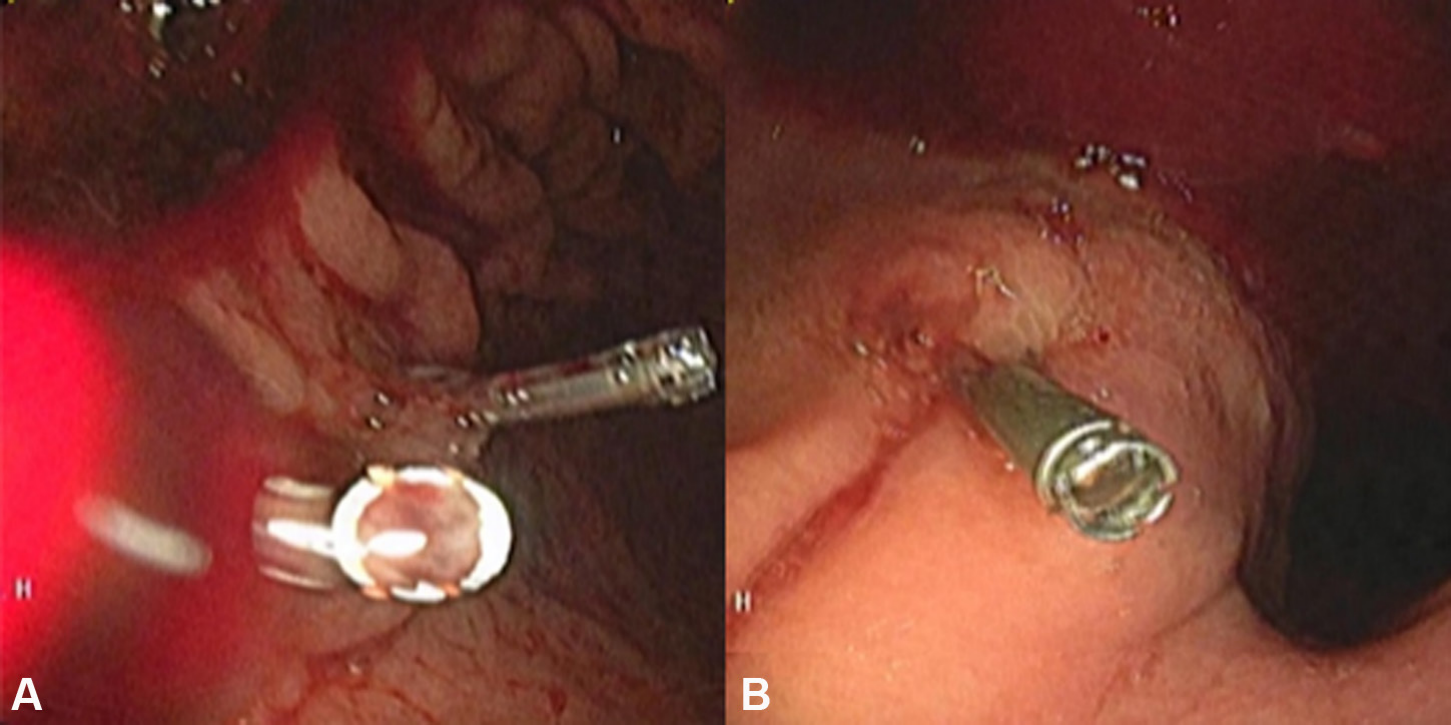
(A) Erosive mucosa in gastric corpus, hemostatic clips. (B) Irregular mucosa in gastric cardia.

**Figure 2.**
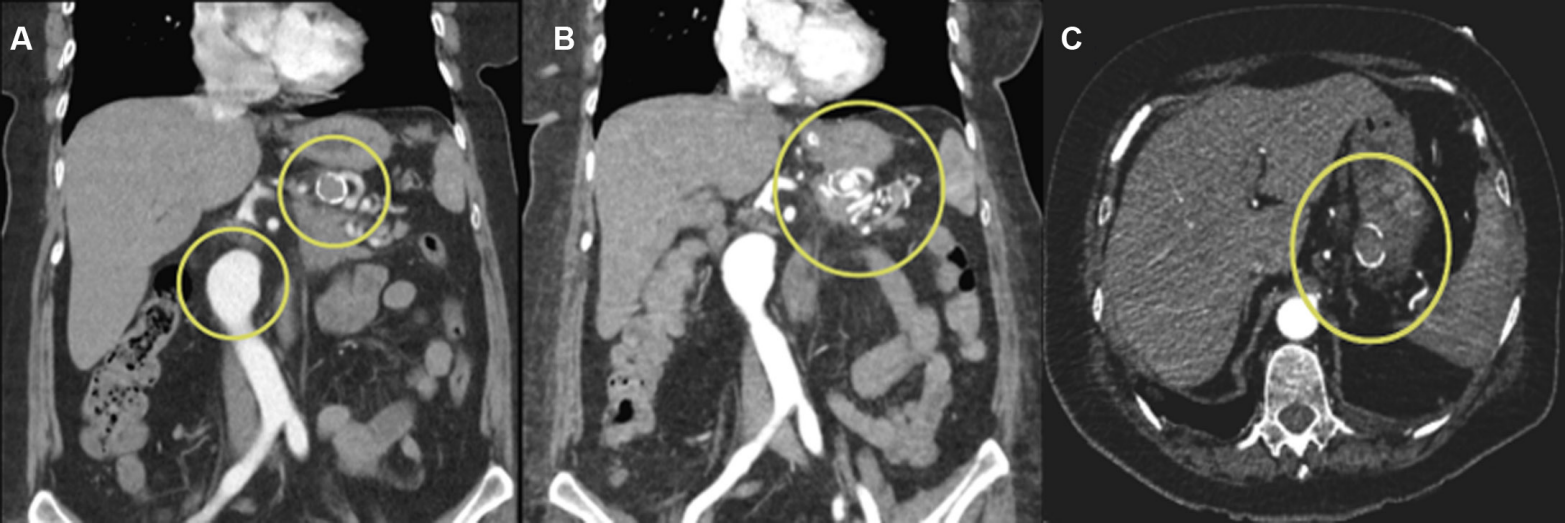
(A) Aneurysmatic dilations in the aorta and splenic artery. (B) Close proximity between the splenic artery aneurysm and the gastric cardia. (C) Complete thrombosis of the aneurysm after coil embolization.

**Figure 3.**
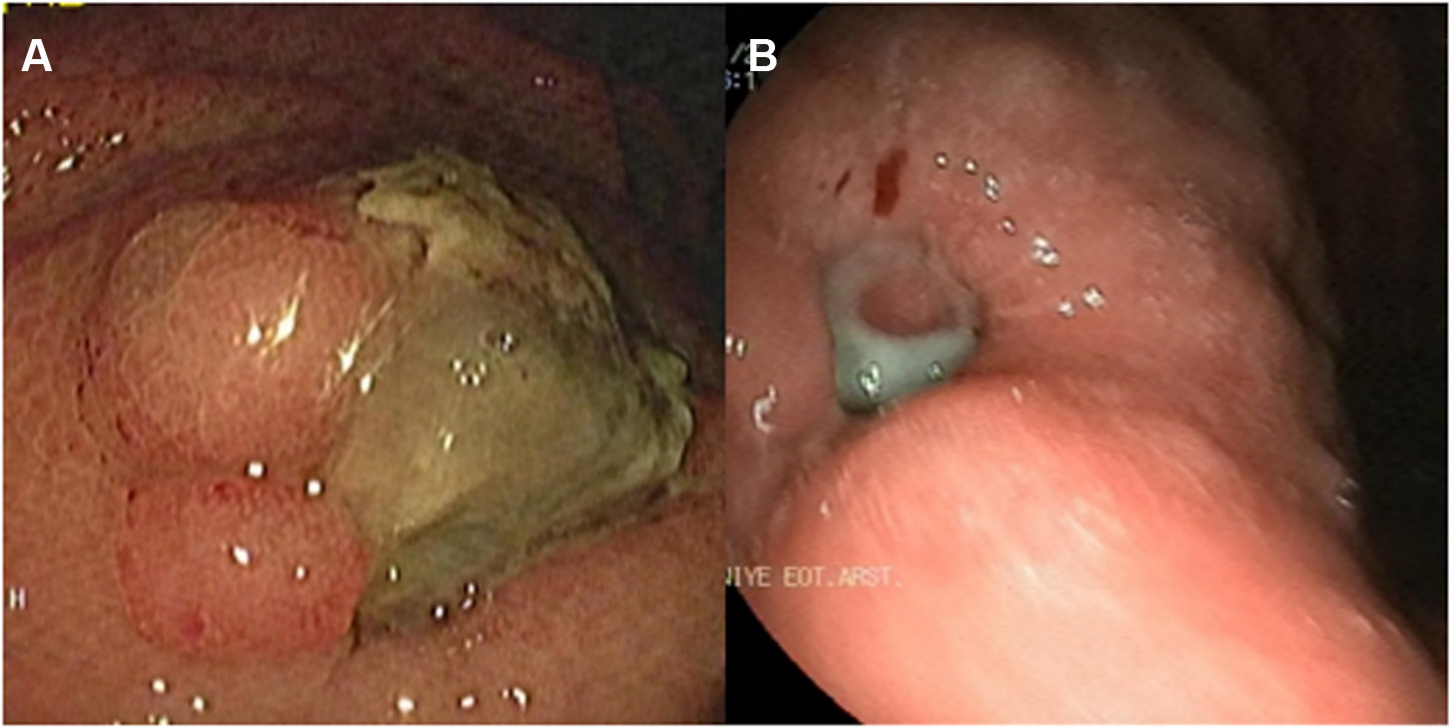
(A) Large exudative ulcer developed in the cardia following coil embolization. (B) Follow-up gastroscopy for the ulcer after 3 months.

## Data Availability

The data that support the findings of this study are available on request from the corresponding author.
